# Observations Pertaining to the Pathology and Treatment of Typhoid Fever

**Published:** 1849-07

**Authors:** W. Matthews

**Affiliations:** Eberle, Ind.


### ARTICLE II.

*Observations Pertaining to the Pathology and Treatment of Typhoid Fever*—By W. MATHEWS, M. D. of Eberle, Ind.

For the last three or four years typhoid fever has been of frequent occurrence in this (Putnam) county.

Respecting its primary cause I need say nothing. I believe that the disease can frequently be traced to a specific contagion, but that in other instances, it originates from other and inscrutable causes. In this respect it has appeared to me to resemble scarlet fever; a disease which, though undeniably contagious, not unfrequently is of spontaneous origin, that is, arises independently of its well known contagious cause.

In conjunction with the consideration of the Pathology of typhoid fever, it is necessary that mention be made of the more prominent of its symptoms, in order to explain certain points therewith connected.

The disease usually makes it attack quite insidiously. The individual about to be attacked complains only of a slight indisposition for perhaps a week or ten days, prior to the evolution of the more unequivocal symptoms of the affection. And it may be that under certain favorable circumstances, like what sometimes happens in scarlatina, typhoid fever produces no structural leisions, and but slight functional disturbances, and the disease thus feebly envolved may elude observation altogether, although the pabulum of life, the blood, may have, in a low degree, been charged wih a poison, which in a higher degree, and more concentrated form, causes the most violent and even fatal commotion of all the vital functions. Therefore in other, and the majority of cases, the constitutional forces are inadequate to resist the working of the cause, the specific poison, when it has once been introduced into the system, the blood perhaps, and results mischevious or even fatal ensue. These results, it is proper to remark, are to be regarded as consequences of the fever, which, as has just been intimated, does occasionally, in my opinion, run its course without complication or being followed by any such untoward effects.

Like all other serious febrile affections, typhoid fever in its course is prone to result in inflammation of one of these structures, the brain, the lungs, or the intestinal tube. Now delirium is by no means an infallible indication of phrenitis, but when it shows itself early in the disease under consideration, and when it is furious, persistent, and accompanies a flushed countenance, heated head and violent carotid pulsation, I believe we may be pretty certain that it exists and that the force of the disease is falling upon the "citadel of life." Patients thus circumstanced, will generally be benefitted by a moderate venesection, by refrigerants to the shaven scalp, and by fearless mercurialization. And such I may add, is the treatment most commonly prescribed. But it has very rarely fallen to my lot to see typhoid fever complicated with cerebral inflammation. In nine cases out of ten, as the disease occurs here, the brain, though its functional aberations be strong and well marked, remains free from organic change.

An error in diagnosis in such cases often leads, I am persuaded, to the gravest consequences, for if the delirium be sympathetic of intestinal specific mucous inflammation, blood letting fails to control it, and reduces the patient, while the mecrury invariably, if often repeated, greatly aggravates its cause.

Pneumonic inflammation is, perhaps, a more frequent accompaniament of typhoid fever, than that of the head. When active, acute inflammation of the pulmonary organs complicate the disease, the tart. antimony, will be found a powerful resolvent agent, but here again, it becomes us to watch the mucous membrane of the alimentary tube. Irreparable mischief must ensue, if we allow a mere sympathetic cough to decoy us from our post. Tart. antimony is a powerful medicine, but in the present disease, I believe, is wholly inadmissable when any evidence of gastro-enteric inflammation exists.

Again, and in at least nine-tenths of all the cases of typhoid fever which have fallen under my immediate observation, the mucous lining of the stomach and bowels, is the seat of the secondary inflammation.

In the treatment therefore of this *abdominal variety* of the disease, it is of the greatest importance to bear this in mind, and to direct our remediate course accordingly. Passire or quiet delirium, a quick wirey pulse, a dry, red tongue, diarrhœa, tympanitis with tenderness, on abdominal pressure being made, are symptoms which ought always to lead us to suspect the existence of lesions of the mucous tissue, but if the diarrhœa prove persistent and will not yield when the appropriate treatment is applied, then, I believe, we need entertain no doubt as to its presence. In cases of this kind the head is not, generally, abnormally hot, and most usually the patient sweats profusely perhaps the fever is paroxysmal, and the delirium more marked at night.

It is this abnormal form of the disorder that post-mortem inspection exhibits traces of pre-existent inflammation of the and mucous folds follicles, known as Peyer's glands. These post-mortem appearances, indeed, have been so constant as to lead some to the conclusion that the disease is essentially a specific inflammation and ulceration of certain portions of the mucous glands and follicles, but there is not evidence sufficient to establish such an opinion.

The treatment in the abdominal variety of the present affection, to be successful, must be expectant. No other articles of food should be allowed than the simplest farinaceous soups, and those only in small quantities. Quiet should be enjoined, and the patient may be allowed cooling vegetable acidulated drinks.

If the vis medicatrix naturæ, be of sufficient strength to carry the patient safely to the termination of that course which the disease is taking, our treatment will consist in leaving it undisturbed. But, on the other hand, if we see the disorder diverging either to the right hand or the left, it is our duty to gently restrain or even coerce it back. But under all circumstances, the natural forces must be trusted with the cure. We are, if possible, to preserve our patient until nature overcomes and expels the disease from the system.

If diarrhœa be present, it probably depends upon ulceration, let us give opium. It is capable of fulfilling various indications in typhoid disease, that is, in the abdominal variety. It not only quiets the nervous symptoms, regulates perspiration, increases the volume, and lessens the frequency of the pulse, but it also, by controling the undue peristaltic movements of intestines, permits the natural forces to overcome and remove the inflammation and ulceraton situated in these organs. It should be administered in small and repeated doses, so as to keep up a constant impression, and, provided it is inadequate to control the diarrhœa, acet. plumb. must be added. Constipation is to be removed by enemata, and, if need be castor oil. But, let me repeat, *above all*, the strictest antiphlogistic regimen must be enjoined.

When collapse ensues, carb. ammonia, camphor, opium, and quinine, with nutritious, unirritating diet are our sheet anchors. Brandy and wine I think inferior to the stimulants above mentioned. In this case we are to sustain life till the reparative powers restore the organic leisions.

With the view of imparting a healing tendency to the ulcerated bowels, Dr. Ross some years ago proposed giving enormous doses of calomel and jalap, supposing the mercury would in its course along the diseased surface act as a tropical agent, but such a practice must be hazardous in the extreme, and none but the fearless will venture to adopt it.

